# Impact of cardiac rehabilitation on cardiovascular event in Korea

**DOI:** 10.1038/s41598-023-46503-3

**Published:** 2023-11-06

**Authors:** In Sun Song, Yu shin Park, Suk-Yong Jang, Jung Mo Nam, Chan Joo Lee, Eun-Cheol Park

**Affiliations:** 1 Department of Public Health, Graduate School, Yonsei University, Seoul, Republic of Korea; 2 Institute of Health Services Research, Yonsei University, Seoul, Republic of Korea; 3 Department of Preventive Medicine, Yonsei University College of Medicine, Seoul, Republic of Korea; 4 Department of Healthcare Management, Graduate School of Public Health, Yonsei University, Seoul, South Korea; 5 Division of Cardiology, Department of Internal Medicine, Severance Hospital, Yonsei University College of Medicine, 50 Yonsei-to, Seodaemun-gu, Seoul, 03722 Republic of Korea; 6 Department of Health Policy, Graduate School of Public Health, Yonsei University, Seoul, South Korea; 7 Department of Preventive Medicine and Institute of Health Services Research, Yonsei University College of Medicine, 50 Yonsei-to, Seodaemun-gu, Seoul, 03722 Republic of Korea

**Keywords:** Interventional cardiology, Health care

## Abstract

This study aimed to evaluate the effects of cardiac rehabilitation (CR) on major adverse cardiac events (MACE) among patients who underwent PCI procedure. We used data from the electronic medical records (EMR) of a tertiary hospital in Seoul, Korea, from January 2014 to February 2020. Data from 2988 patients who had experienced their first acute coronary syndrome (ACS) and had undergone percutaneous coronary intervention (PCI) were included during the study period. we classified patients into CR participants and non-participants based on their participation in the cardiac rehabilitation (CR) program within 30 days after discharge. And the outcome was the incidence of myocardial infarction (MI) and stroke within 1 year after discharge. The association between participation in CR and risk of developing MACE was evaluated using the Cox proportional hazards model. Patients who achieved CR after undergoing PCI were at a lower risk of developing MI (HR 0.68, CI 0.53–0.86). There was no significant association between participation in CR and the incidence of stroke. Among patients who had more than three stenotic vessels, the risk of developing MI within 1 year of discharge was reduced in CR users compared to non-users (3 or more stenosis vessels: HR 0.55, CI 0.35–0.86). Among patients who used two and more stents during PCI procedures, the risk of developing MI within 1 year of discharge was reduced in CR users compared to non-users (2 and more stents: HR 0.54, CI 0.35–0.85). Among people diagnosed with ACS and receiving PCI, patients who participated in CR within one month of discharge reduced risk of developing MI. Our study reinforced the current evidence on the effect of CR among patients receiving PCI and presented the expansion and enhancement of the CR program.

## Introduction

Heart disease is a major cause of premature mortality and an important cause of disability globally^1,2^. In Korea, heart disease is the second major cause of death. Ischemic heart disease, which accounts for approximately half of the deaths from all heart diseases, increased from 12,893 in 2009 to 13,699 in 2019^3^. According to the World Heart Federation, the global cost of cardiovascular disease (CVD) in 2010 was approximately US $863 billion, which is expected to rise to more than US $1 trillion by 2030^4^.

Most treatments for severe heart disease involve cardiac surgery or medication. Postoperative complications include pulmonary complications, delirium, and arrhythmias^5–7^. Furthermore, restenosis of heart disease occurs in approximately 30–50% of patients undergoing percutaneous coronary intervention^8^. These complications are linked to a long period of hospitalization, increased adverse events, and higher healthcare costs^9–12^.

To minimize and prevent the possibility of disease recurrence after percutaneous coronary intervention, the 2011 American Heart Association recommends participation in pre-discharge cardiac rehabilitation (CR) at the Class I level and published the relevant guideline^13^. The goal of CR is to improve the preoperative and postoperative status of patients undergoing CVD^14^. CR is a program designed to minimize recurrence and related complications of CVD through proper medication, improvement of lifestyle for diabetes, high blood pressure, hyperlipidemia, exercise, diet, smoking cessation, and stress^15^.

Previous studies have shown that CR programs for coronary artery disease patients have reduced mortality and increased survival rates through exercise, diet, smoking cessation, weight control, diabetes, and hypertension management^16–18^. As the effectiveness of CR was proven, the demand for heart rehabilitation increased. The problem of economic burden, which was one of the factors for underutilizing of CR, was resolved when CR services after IHD started being reimbursed as medical treatment benefits since February 2017. Currently, 111 countries worldwide have implemented CR^19^.

In Korea, few studies have evaluated the effectiveness of CR using clinical data, and few studies have considered the duration of the event. This study aimed to evaluate the effects of CR on major adverse cardiac events (MACE) caused by myocardial infarction, stroke for 1 year after discharge, using patient data from a tertiary hospital in Seoul. We hypothesize that CR is necessary to prevent recurrence and deterioration of patients with coronary artery disease.

## Method

### Data

We used data from the electronic medical records (EMR) of a tertiary hospital in Seoul, Korea, from January 2014 to February 2020. The EMR included clinical information of eligible patients, such as demographics, past medical history, vital signs, laboratory findings, diagnosis of acute coronary syndrome (ACS), level of education, performance status, surgery type, and procedure of surgery. The data was encrypted to safeguard personal information.

### Participants

**Figure 1:**
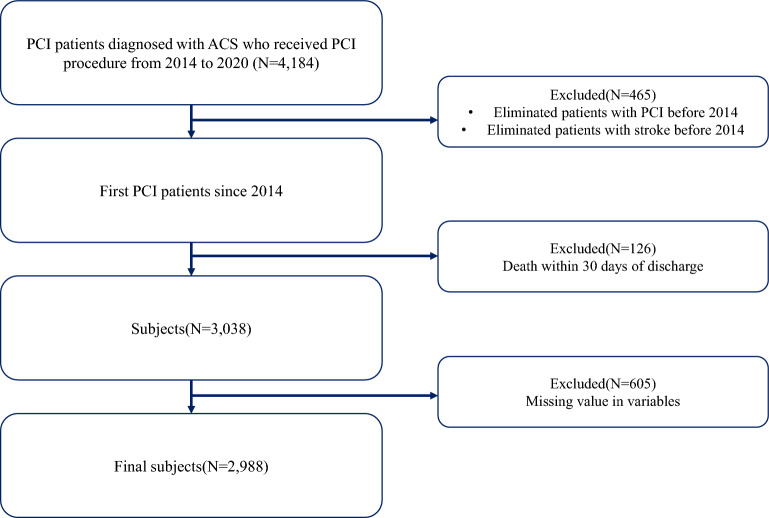
Flow chart of the study.

We extracted individuals who had diagnosed acute coronary syndrome (ACS) and undergone PCI procedures from January 2014 to February 2020. Consequently, those who had received PCI procedures before 2014 were excluded, and individuals diagnosed with stroke before 2014 were also excluded to create a homogeneous population. Additionally, individuals covered by medical aid program were excluded due to different healthcare systems in South Korea, and only those with national health insurance were included. Furthermore, individuals who died within 30 days after discharge were also excluded (Fig. 1). Finally, we extracted data pertaining to 2988 patients.

### Utilization of Cardiac rehabilitation

In this study, individuals who engaged in the cardiac rehabilitation (CR) program within 30 days of their discharge following PCI were categorized as CR participants. The CR program comprised three components: education, exercise, and evaluation, and patients' involvement in any of these aspects was identified by fee codes. However, there was no patients who participated in evaluation program. CR utilization was characterized as a patient's involvement in one or more of the CR program elements (education, exercise, both) during the initial 30 days following their PCI discharge.

### Outcome

The outcome was the incidence of major adverse cardiovascular event (MACE) within 1 year after discharge. Many studies have used MACE for evaluating the effect of CR as an indicator of one result rather than measuring the effect for each result^20,21^. In our study, MACE is defined as the incidence of myocardial infarction (MI), stroke which are important prognostic factors that may appear in heart disease patients as major heart events. These are indicators commonly identified in various studies to confirm the effect of CR^22^. We identified participants with a diagnosis of myocardial infarction (MI) using ICD-10 codes I21, I22, I23, I25.2, and I25.5, as well as those with a diagnosis of stroke using codes I60 through I69. The occurrence of MACE after discharge of the study subjects was monitored for 1 year using diagnosis codes and EMR. Patients who were re-hospitalized under the code name of MI and stroke within 1 year were identified by follow-up observation based on the date of initial discharge.

### Variables

We controlled for covariates, such as sociodemographic, socioeconomic, health information, and clinical factors. Sociodemographic factors included sex (male and female), age (less than 60, 60–69, and over 70 years), and education level (elementary or middle school, high school). Socioeconomic factors included difficulty paying medical bills (yes or no). Health information factors included smoking status (current, ex, non), BMI (< 25, ≥ 25), regular exercise (yes or no), and past medical history (hypertension, dyslipidemia, cancer, chronic kidney disease). We considered the five clinical factors; When a patient was initially diagnosed with their first ACS, the diagnosis was categorized as unstable angina (UA, I20.0, I24.0), which was identified by ICD-10 codes, and this was classified separately from MI (I21, I22, I23, I25.2, I25.5). and we divided into individuals based on their use of stents during PCI procedures, categorizing them as “stent operators” if they used stents at least once and as “ballooning operators” if they exclusively performed ballooning procedures. Additionally, a small number of cases involving percutaneous thrombectomy and embolectomy were included in the ballooning procedure category. The corresponding ICD-10 codes were included in the supplementary table. We also included the number of vascular stenosis (1, 2, or 3 or more), the number of stent (0, 1, 2 or more) and target vessel of PCI procedure(left main or left anterior descending artery, others) during PCI procedure according to procedure of surgery note. We intend to use the number of stenosis vessel, stent and target vessel as proxy indicators for severity of health status.

### Statistical analysis

The chi-squared test was used to determine the significant differences in variables between participants who died and those who did not. Statistical significance was set at P-value < 0.05. A Cox proportional hazards model was used to evaluate the association between participation in CR and incidence of MACE. The results were reported using hazard ratios (HRs) and 95% confidence intervals (CIs) for the risk of MACE. Each patient’s index date was the discharge date after the first PCI during the study period. And discharge dates and admission dates only include the year and month. Therefore, we assumed an arbitrary day to be the 1st of the month in our dataset. Additionally, the incidence of the event was analyzed by monitoring the index data for 6 months, 2 years, and 3 years. The data were analyzed using SAS 9.4 (SAS Institute Inc; Cary, North Carolina).

### Ethics declarations

The Institutional Review Board of Yonsei University Health System approved this study (approval number: 4-2022-0452) and waived the requirement for informed consent as The Korean Vital Statistics database consists of anonymized, and unidentified patient data. And the study was conducted according to the tenets of the 2013 Declaration of Helsinki.

### Ethical approval

The requirement for informed consent was waived by Yonsei University Health System institutional review board (4–2022-0452) as The Korean Vital Statistics database consists of anonymized, and unidentified patient data. We assert that all procedures contributing to this work comply with the ethical standards of the relevant national and institutional committees on human experimentation and with the Helsinki Declaration of 1975, as revised in 2008.

## Results

Table 1 presents the general characteristics of the baseline study participants. A total of 2988 participants were diagnosed ACS and undergone PCI procedures during study period. Among the patients who underwent PCI, 339 (11.3%) had incident MI within 1 year of discharge, 31 (1%) developed stroke within 1 year of dischrge. Out of 2988 participants, 1156 (38.7%) participated in the CR program within 30 days after discharge.

**Table 1:** General characteristics of the baseline study population.

**Variables**			MI				*P-value*	Stroke				*P-value*
	Total		Yes		No			Yes		No		
	N	%	N	%	N	%		N	%	N	%	
Total	2988	100.0	339	11.3	2,649	69.7		31	1.0	2,957	99.0	
Participation in Cardiacrehabilitation							< 0.0001					0.159
Yes	1156	38.7	97	8.4	1059	91.6		8	0.7	1148	99.3	
No	1832	61.3	242	13.2	1590	86.8		23	1.3	1809	98.7	
Sex							0.944					0.596
Male	2243	75.1	255	11.4	1988	88.6		22	1.0	2221	99.0	
Female	745	24.9	84	11.3	661	88.7		9	1.2	736	98.8	
Age		0.0					0.160					0.003
-59	1060	35.5	109	10.3	951	89.7		4	0.4	1056	99.6	
60–69	917	30.7	100	10.9	817	89.1		8	0.9	909	99.1	
70-	1011	33.8	130	12.9	881	87.1		19	1.9	992	98.1	
Educational level							0.055					0.027
Elementary or middle school	821	27.5	108	13.2	713	86.8		14	1.7	807	98.3	
High school, College or above	2167	72.5	231	10.7	1936	89.3		17	0.8	2150	99.2	
Difficulty paying medical bill								0.0				0.265
Yes	152	5.1	13	8.6	139	91.4		3	2.0	149	98.0	
No	2836	94.9	326	11.5	2510	88.5		28	1.0	2808	99.0	
Smoking status							0.092					0.031
Current smoker	732	24.5	71	9.7	661	90.3		3	0.4	729	99.6	
Ex-smoker	795	26.6	105	13.2	690	86.8		14	1.8	781	98.2	
Non-smoker	1461	48.9	163	11.2	1298	88.8		14	1.0	1447	99.0	
Drinking status							0.029					0.001
Current drinker	1171	39.2	115	9.8	1056	90.2		5	0.4	1166	99.6	
Ex-drinker	405	13.6	59	14.6	346	85.4		11	2.7	394	97.3	
Non-drinker	1412	47.3	165	11.7	1247	88.3		15	1.1	1397	98.9	
BMI (kg/m^2^)							0.595					0.809
< 25	1671	55.9	185	11.1	1486	88.9		18	1.1	1653	98.9	
≥ 25	1317	44.1	154	11.7	1163	88.3		13	1.0	1304	99.0	
Regular exercise							0.376					0.566
Yes	500	16.7	51	10.2	449	89.8		4	0.8	496	99.2	
No	2488	83.3	288	11.6	2200	88.4		27	1.1	2461	98.9	
Risk factors												
Hypertension							0.039					0.048
No	622	20.8	56	9.0	566	91.0		2	0.3	620	99.7	
Yes	2366	79.2	283	12.0	2083	88.0		29	1.2	2337	98.8	
Diabetes							0.006					0.311
No	1619	54.2	160	9.9	1459	90.1		14	0.9	1605	99.1	
Yes	1369	45.8	179	13.1	1190	86.9		17	1.2	1352	98.8	
Dyslipidemia							0.343					0.527
No	513	17.2	52	10.1	461	89.9		4	0.8	509	99.2	
Yes	2475	82.8	287	11.6	2188	88.4		27	1.1	2448	98.9	
Cancer							0.029					0.631
No	2614	87.5	284	10.9	2330	89.1		28	1.1	2586	98.9	
Yes	374	12.5	55	14.7	319	85.3		3	0.8	371	99.2	
Chronic kidney							< 0.0001					0.001
No	1685	56.4	147	8.7	1538	91.3		8	0.5	1677	99.5	
Yes	1303	43.6	192	14.7	1111	85.3		23	1.8	1280	98.2	
Cardiac diagnosis							0.591					0.162
UA	1625	54.4	189	11.6	1436	88.4		13	0.8	1612	99.2	
MA	1363	45.6	150	11.0	1213	89.0		18	1.3	1345	98.7	
Type of procedure							< 0.0001					< .0001
PCI (Stent)	2875	96.2	313	10.9	2562	89.1		28	1.0	2847	99.0	
PTCA (Balloon)	113	3.8	26	23.0	87	77.0		3	2.7	110	97.3	
Target vessel							0.000					0.028
LM and LAD	1735	58.1	166	9.6	1569	90.4		12	0.7	1723	99.3	
Others	1253	41.9	173	13.8	1080	86.2		19	1.5	1234	98.5	
Number of stenosis vessels								0.0				< .0001
1	1258	42.1	101	8.0	1157	92.0		6	0.5	1252	99.5	
2	946	31.7	120	12.7	826	87.3		12	1.3	934	98.7	
3 ≤	784	26.2	118	15.1	666	84.9		13	1.7	771	98.3	
Number of Stents							0.000					0.187
None	108	3.6	25	23.1	83	76.9		3	2.8	105	97.2	
1	2010	67.3	212	10.5	1798	89.5		19	0.9	1991	99.1	
2 ≤	870	29.1	102	11.7	768	88.3		9	1.0	861	99.0	

**Figure 2:**
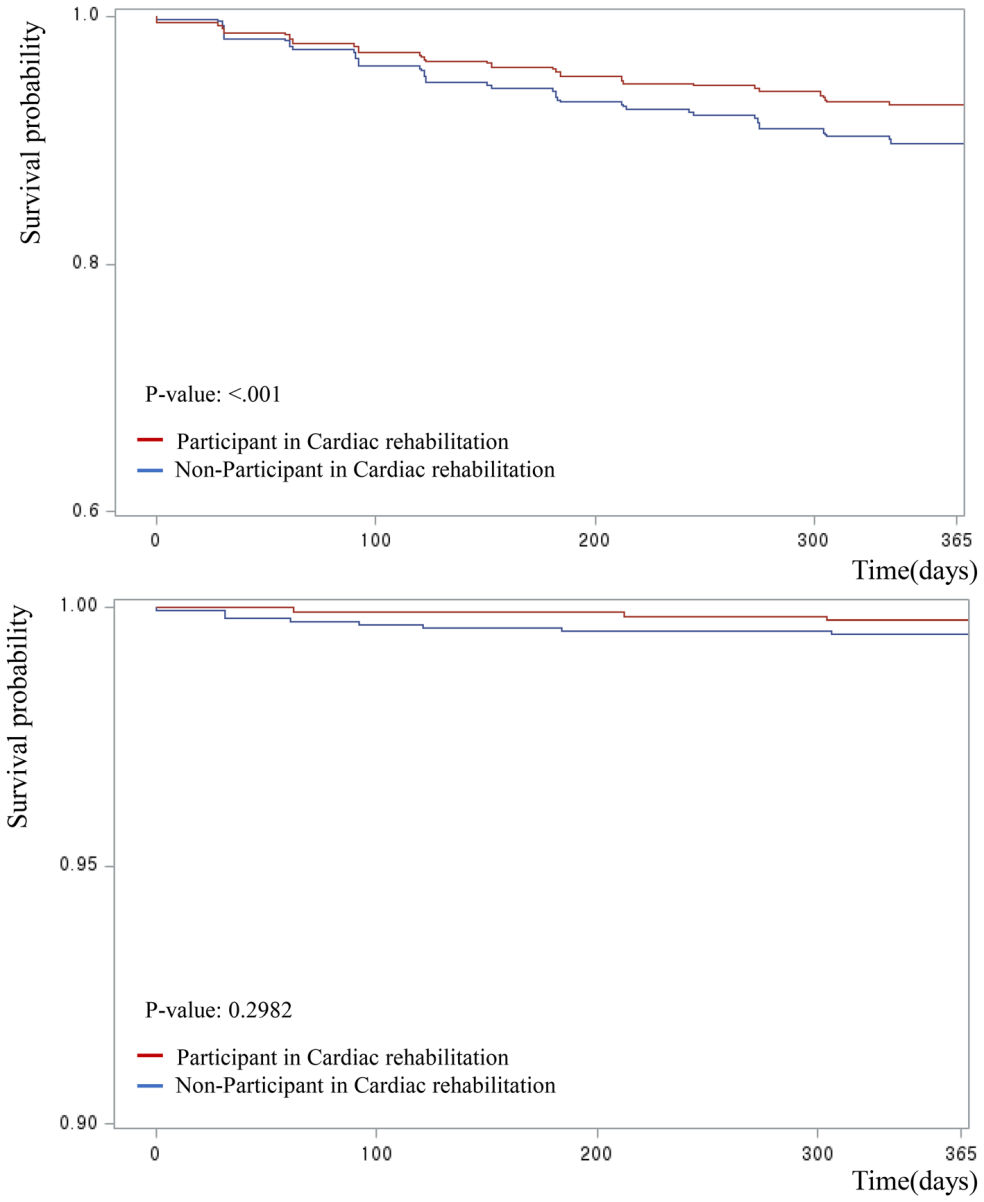
Kaplan–Meier survival curves for participants in cardiac rehabilitation in 1-year occurrence of readmission for MI and stroke.

Figure 2 presents the Kaplan–Meier survival curves comparing the incidence of MI and stroke between CR users and non-users. Vertical lines indicate the non-incidence probability of MI and stroke, and horizontal lines indicate the monitoring days. Patients who participated in CR less frequently developed MI and stroke (MI: p < 0.0001, Stroke: p < 0.2982).

Table 2 reports the findings of Cox proportional hazards regression analysis regarding the association between participation in CR and incidence of MI and stroke after adjusting for the abovementioned covariates. Patients who utilized CR after undergoing PCI were at lower risk of developing MI (HR 0.68, CI 0.53–0.86) compared to CR non-user, and there was no significant association between participation in CR and incidence of stroke (HR 0.74, CI 0.33–1.70).

**Table 2:** Association between participation in cardiac rehabilitation and 1-year occurrence of MACE.

Variables	MI		Stroke	
	HR	95% CI	HR	95% CI
Participation in cardiac rehabilitation				
Yes	0.68	(0.53–0.86)	0.74	(0.33–1.70)
No	1.00		1.00	
Sex				
Male	1.00		1.00	
Female	1.01	(0.73–1.39)	1.36	(0.47–3.99)
Age				
50–59	1.00		1.00	
60–69	0.87	(0.65–1.16)	1.74	(0.51–5.97)
70-	0.89	(0.66–1.20)	2.59	(0.79–8.48)
Educational level				
Elementary or middle school	1.00		1.00	
High school, College or above	0.84	(0.64–1.09)	0.74	(0.33–1.65)
Difficulty paying medical bill				
Yes	0.67	(0.39–1.18)	1.98	(0.59–6.70)
No	1.00		1.00	
Smoking status				
Current smoker	0.93	(0.67–1.29)	0.74	(0.18–3.02)
Ex-smoker	1.13	(0.83–1.52)	1.83	(0.67–5.06)
Non-smoker	1.00		1.00	
Drinking status				
Current drinker	0.94	(0.71–1.24)	0.69	(0.22–2.13)
Ex-drinker	1.17	(0.83–1.64)	2.71	(1.01–7.26)
Non-drinker	1.00		1.00	
BMI (kg/m^2^)				
< 25	1.00		1.00	
≥ 25	1.03	(0.83–1.29)	0.92	(0.45–1.91)
Regular exercise				
Yes	1.00		1.00	
No	1.13	(0.84–1.53)	1.33	(0.46–3.88)
Risk factors				
Hypertension				
No	1.00		1.00	
Yes	1.12	(0.84–1.51)	2.45	(0.56–10.67)
Diabetes				
No	1.00		1.00	
Yes	0.75	(0.55–1.02)	0.45	(0.19–1.08)
Dyslipidemia				
No	1.00		1.00	
Yes	1.04	(0.77–1.41)	1.60	(0.54–4.75)
Cancer				
No	1.00		1.00	
Yes	1.22	(0.90–1.64)	0.36	(0.11–1.26)
Chronic kidney				
No	1.00		1.00	
Yes	1.89	(1.38–2.60)	4.14	(1.51–11.29)
Cardiac diagnosis				
UA	1.00		1.00	
MA	1.00	(0.80–1.25)	1.66	(0.79–3.52)
Type of procedure				
PCI (Stent)	1.00		1.00	
PTCA (Balloon)	1.41	(0.54–3.68)	1.46	(0.02–88.49)
Target vessel				
LM and LAD	0.71	(0.58–0.89)	0.51	(0.24–1.06)
Others	1.00		1.00	
Number of stenosis vessels				
1	1.00		1.00	
2	1.51	(1.15–1.98)	2.12	(0.79–5.73)
3 ≤	1.71	(1.28–2.27)	2.28	(0.83–6.24)
Number of Stents				
None	1.00		1.00	
1	0.66	(0.25–1.76)	0.50	(0.01–30.38)
2 ≤	0.65	(0.24–1.76)	0.50	(0.01–31.66)

Subgroup analysis was conducted to evaluate the effect of participation in CR and the characteristics of the clinical procedure on the risk of developing MI and stroke (Table3). Among patients with UA, the risk of MI within 1 year of discharge was lower in CR users than in non-users (HR 0.56, CI 0.39–0.79). Among patients who had more than three stenotic vessels, the risk of developing MI within 1 year of discharge was reduced in CR users compared to non-users (3 or more stenosis vessels: HR 0.55, CI 0.35–0.86). Among patients who used two and more stents during PCI procedures, the risk of developing MI within 1 year of discharge was reduced in CR users compared to non-users (2 and more stents: HR 0.54, CI 0.35–0.85).

**Table 3:** Subgroup analysis stratified by independent variables.

Variables	MI			Stroke		
	Participation in cardiac rehabilitation					
	No	Yes		No	Yes	
	HR	HR	CI	HR	HR	CI
Cardiac diagnosis						
UA	1.00	0.56	(0.39–0.79)	1.00	0.42	(0.09–1.97)
MI	1.00	0.84	(0.60–1.18)	1.00	1.03	(0.36–2.99)
Type of procedure						
PCI (Stent)	1.00	0.70	(0.54–0.89)	1.00	0.84	(0.36–1.97)
PTCA (Balloon)	1.00	0.46	(0.12–1.78)	–	–	–
Target vessel						
LM and LAD	1.00	0.66	(0.47–0.94)	1.00	0.70	(0.18–2.78)
Others	1.00	0.66	(0.47–0.93)	1.00	0.82	(0.28–2.40)
Number of stenosis vessels						
1	1.00	0.71	(0.46–1.09)	1.00	1.47	(0.16–13.56
2	1.00	0.72	(0.49–1.07)	1.00	1.02	(0.24–4.41)
3 ≤	1.00	0.55	(0.35–0.86)	1.00	0.94	(0.24–3.78)
Number of Stents						
None	1.00	0.55	(0.14–2.25)	–	–	–
1	1.00	0.79	(0.58–1.06)	1.00	1.36	(0.51–3.65)
2 ≤	1.00	0.54	(0.35–0.85)	1.00	0.12	(0.01–1.35)

Table 4 shows the result of the association between participation in type of CR and incidence of MI and stroke. Patients who participated in the education program in CR had a lower risk of developing MI (HR 0.42, CI 0.31–0.59), and patients who participated in both the education and exercise programs in CR had a reduced risk of MI (HR 0.42, CI 0.27–0.65).

**Table 4:** The results of subgroup analysis stratified by type of cardiac rehabilitation.

Variables		MI		Stroke	
		HR	95% CI	HR	95% CI
Participation in cardiac rehabilitation					
Type of cardiac rehabilitation	Education				
	Yes	0.42	(0.31–0.59)	0.26	(0.06–1.10)
	No	1.00		1.00	
	Exercise				
	Yes	0.83	(0.64–1.07)	0.92	(0.37–2.31)
	No	1.00		1.00	
	Education & exercise				
	Yes	0.42	(0.27–0.65)	–	–
	No	1.00		–	

## Discussion

In this study, we investigated the association between participation in CR and the incidence of MI and stroke among people diagnosed with ACS and receiving PCI. Few studies have investigated the effectiveness of CR using detailed clinical data on PCI procedures after the patients’ first ACS diagnosis. Participation in CR among patients who underwent a PCI procedure reduced the risk of developing MI within 1 year after discharge, but it was not associated with an increased risk of developing stroke. Also, participation in CR reduced risk of developing MI to a greater extent in patients who have multiple stenotic vessels and numerous stents.

Our results showed that patients who participated in CR within a month of discharge had a 32% lower probability of developing MI than those who did not. Previous studies have demonstrated the association between utilizing CR and MACE, which are generally in similar result to our study^23–26^. However, our study could not find evidence that participation in CR reduced the risk of stroke. In the Kaplan–Meier survival curve, there were significant differences between CR users and non-users by log-rank test. However, when we adjusted the covariates, there was no significant difference between CR users and non-users. Other studies have demonstrated an association between participation in cardiac rehabilitation (CR) and a reduced risk of stroke. These studies included participants with a prior history of stroke occurring before ACS events and adjusted their analyses accordingly. In contrast, our study adopted a different approach by excluding individuals with a history of stroke. This deliberate design choice introduces a potential divergence in results between our study and those that encompassed stroke history, warranting consideration when interpreting the findings^27,28^.

In our study, there was no significant difference in the risk of developing MI within 1 year after discharge among individuals diagnosed with UA or MI who underwent PCI procedures. However, participation in CR among UA patients significantly reduced the risk of MI occurrence within 1 year after discharge. However, participation in CR among MI patients was not associated with the risk of MI occurrence within 1 year after discharge. When individuals were diagnosed with ACS and underwent PCI, a higher number of stenosis vessels was associated with an increased risk of MI occurrence within 1 year after discharge. However, among patients with a higher number of stenosis vessels at the time of PCI, participation in cardiac rehabilitation within one month of discharge was associated with a significant reduction in the risk of MI occurrence within 1 year after discharge. Few studies have evaluated the effectiveness of CR in relation to the severity of patients with heart disease. Ejection fraction (EF) is the most important parameter in evaluating the severity of heart disease, and studies have shown that there is no association between heart rehabilitation and death or other events in patients with heart failure with reduced ejection fraction (HFrEF) or severely reduced EF^29–31^. However, there are still ongoing discussions about eligible participants of CR, and first-class recommendations target people with less than 35–40% EF^32^. In our study, due to data limitations, we were unable to ascertain the level of EF. However, we were able to assess the characteristics of patients' ACS, which may provide a basis for determining eligibility for participation in CR.

In recent studies, the effects of cardiac rehabilitation have been studied based on the patients' conditions, including obesity, hospital-acquired functional impairment, multimorbidity, and more. Among patients who participated in cardiac rehabilitation, patients with higher lean body mass (LBM) and body mass index (BMI) showed an increased risk of major adverse cardiovascular events (MACE), especially in the presence of multimorbidity^33,34^. Additionally, in the group of patients who experienced post-surgical hospital-acquired functional decline, participation in cardiac rehabilitation present that reducing the risk of MACE^34^. Our study examined the effectiveness of cardiac rehabilitation based on the disease status of patients at the time of PCI procedure, providing valuable insights into its impact on patient outcomes. Patients exhibiting diverse health conditions might be considered for personalized therapeutic approaches within cardiac rehabilitation.

In Korea, the government has established and operated a policy to strengthen health insurance coverage since 2005 to mitigate the economic burden of serious diseases (cancer, heart disease, cerebrovascular disease) for reducing out-of-pocket expenditure and converting benefits for non-benefit items. The CR program in Korea has been covered by the National Health Insurance since February 2017. The CR participation rate among cardiac patients in Korea ranged from 0.0 to 6.4% and was also low in hospitals actively delivering CR (14–35%)^35,36^. Because we studied data from an institution that is one of the largest providers of CR in Korea, the participation rate in CR was 38.7% in our study. Worldwide, only 38.8% of countries have CR programs^19,37,38^. Despite these benefits and recommendations in clinical practice guidelines, CR was grossly underused compared with other medical treatments for patients with CVD. Various studies and service providing systems are needed to promote CR.

This study has a few limitations. First, it was a retrospective study performed in a single-tertiary university hospital, lacking external validity. However, our study has the strengths of including a patient group with a history of ARS that is a high-risk group for MACE, having detailed long-term follow-up surveillance data, and analyzing detailed PCI procedures and status of ACS, which are related to the incidence of MACE. This can strengthen the accuracy of our findings. However, further studies with larger sample sizes, longer follow-up periods, and more events are needed to validate our findings.

## Conclusions

Among people diagnosed with ACS and receiving PCI, patients who participated in CR within one month of discharge reduced risk of developing MI. Our study reinforced the current evidence on the effect of CR among patients receiving PCI and presented the expansion and enhancement of the CR program.

### Supplementary Information

Supplementary Tables.

## Data Availability

The data that support the findings of this study are available on request from the corresponding author.
