# Supplementary material for: Impact of cardiac rehabilitation on cardiovascular event in Korea
Source: Sci Rep. 2023 Nov 6;13:19146. doi: 10.1038/s41598-023-46503-3 (PMC10628201; doi:10.1038/s41598-023-46503-3)
Supplement: Supplementary file 1 — Supplementary Tables. [file 41598_2023_46503_MOESM1_ESM.docx]

Supplementary table. diagnosis codes (ICD-10)

| **ICD-10 code** | |
| --- | --- |
| I20.0 | Unstable angina |
| I24.0 | Coronary thrombosis not resulting in myocardial infarction |
| I21 | Acute myocardial infarction |
| I22 | Subsequent myocardial infarction |
| I23 | Certain current complications following acute myocardial infarction |
| I25.2 | Old myocardial infarction |
| I25.5 | Ischemic cardiomyopathy |

Supplementary table. Procedure code(ICD-10)

| **ICD-10 code** | |
| --- | --- |
| Percutaneous Transcatheter Coronary Intervention | M6561, M6562, M6563, M6564, M6565, M6566, M6567 |
| percutaneous transluminal coronary angioplasty | M6551, M6552, M6553, M6554 |
| percutaneous thrombectomy | M6571 |
| percutaneous  embolectomy | M6638 |
| CABG | O1640, O1641, O1647, O1648, O1649, OA640, OA641, OA647, OA648, O1649, O0170, O0171 |
